# Prediction of acute mountain sickness occurring at 4554 m using overnight pulse oximetry from lower altitude: A pilot study

**DOI:** 10.1113/EP093777

**Published:** 2026-09-30

**Authors:** Luke Cutts, Kim Ashdown, Ciaran Simpkins, Will Trender, Sarah Clarke, John Delamere, Samuel J. E. Lucas, Kelsey Elizabeth Joyce

**Affiliations:** ^1^ Salford Royal NHS Trust Salford UK; ^2^ Medical School for Birmingham Medical Research Expeditionary Society University of Birmingham Birmingham UK; ^3^ Occupational Performance Research Group University of Chichester Chichester UK; ^4^ Medical School University of Birmingham Birmingham UK; ^5^ Restorative Neurosciences Imperial College London London UK; ^6^ Anaesthesia & Intensive Care Medicine Royal Blackburn Teaching Hospital, NHS Trust Blackburn UK; ^7^ School of Sport, Exercise and Rehabilitation Sciences University of Birmingham Birmingham UK; ^8^ Human Integrative Physiology Lab Mayo Clinic Arizona Scottsdale Arizona USA

**Keywords:** acute mountain sickness, high altitude, hypoxia, pulse oximetry

## Abstract

Acute mountain sickness (AMS) affects many individuals ascending to high altitude annually, carrying potential morbidity and mortality risks (e.g., high‐altitude cerebral oedema). The objective of this pilot study was to record overnight pulse oximetry during ascent to 4554 m and determine whether: (1) extracted overnight biomarkers had any relationship to AMS; and (2) AMS at 4554 m could be predicted by oximetry biomarkers using classification models trained/validated on overnight data from lower altitudes. Twenty lowlanders (five females, 36.8 ± 18.5 years old) completed a 4 day ascent to 4554 m, where AMS status was determined by clinical examination. Oximetry was recorded continuously each night, with >40 biomarkers extracted and compared between AMS and non‐AMS. Exploratory classification models (5‐fold cross‐validation) were tested for nightly data with a systematic approach to feature selection. Model performances were evaluated based on predictions of AMS at 4554 m. A significant effect of ascent was observed for many overnight biomarkers; however, no effect of AMS status was evident, nor were any differences observed between AMS and non‐AMS for any overnight biomarkers during ascent. AMS at 4554 m was most accurately predicted from overnight recordings collected at 2600 m (accuracy, 100%; true positive rate, 100%) and 3647 m (accuracy, 80%; true positive rate, 90.9%) using *k*‐nearest neighbour and support vector machine classification models. Biomarkers were extracted from overnight oximetry recordings, with limited differences observed between AMS and non‐AMS. In conclusion, AMS at 4554 m can be predicted from overnight oximetry using machine learning; however, these preliminary findings need confirmation in a larger cohort, with additional investigation into the most clinically relevant biomarkers.

## Abstract (highlights)

- **What is the central question of this study?**
  Can overnight oximetry be used to predict acute mountain sickness during ascent to high altitude?
- **What is the main finding and its importance?**
  Classification models applied to overnight pulse oximetry biomarkers can be used to predict acute mountain sickness prospectively and prior to the onset of clinical presentation, as shown in this pilot study. Predictions of acute mountain sickness using such classification models applied to overnight pulse oximetry might be optimised based on location/altitude of overnight recording and feature selection.

## 1 INTRODUCTION

Acute mountain sickness (AMS) affects a large proportion of individuals ascending to high altitude each year and, if left untreated, can progress to more severe illness (e.g., high‐altitude cerebral oedema) that can require emergency rescue or cause fatality. Despite this well‐established observation and outcome, predicting the onset of AMS prior to any detection of clinical symptoms remains elusive. We previously assessed the utility of overnight versus morning pulse oximetry (K. E. Joyce et al., 2024) and, in a recent systematic review, also highlighted and discussed the potential use of overnight oximetry as detector and predictor of AMS during ascent to altitude (Goves et al., 2024). Nevertheless, it was clear that further investigations are warranted to determine which physiological biomarkers from overnight oximetry are relevant for detecting AMS and whether such biomarkers are predictive of AMS.

The objective of the present pilot study was to collect overnight oximetry data during ascent to high altitude with the following aims: (1) to assess the feasibility of extracting a range of physiological biomarkers from overnight oximetry data; and (2) to determine whether these biomarkers differed between AMS and non‐AMS. Exploratory analysis included the preliminary testing/validation of classification models based on overnight oximetry biomarkers (collected before AMS symptoms developed) for the prediction AMS occurring at higher altitudes. Performances of these nightly models for their predictions of AMS occurring at the summit (at 4554 m, Margherita Hut) were also evaluated.

We hypothesised that biomarkers could be extracted successfully from overnight recordings and that several biomarkers might exhibit significant differences between AMS and non‐AMS before reaching or at the summit. Likewise, we expected that several of the differing biomarkers might also be selected for inclusion in the preliminary classification model.

## 2 MATERIALS AND METHODS

### 2.1 Ethical approval

Ethical approval was granted by the University of Birmingham (ERN 21‐0216) for this pilot study. The study was conducted in accordance with the *Declaration of Helsinki*, 2018 (except for registration in a database). Methodology was conducted/reported in accordance with the STAR reporting guidelines wherever possible, which are specific to altitude research (Brodmann Maeder et al., 2018). Written informed consent was obtained from all individuals prior to participation.

### 2.2 Study design and participants

Twenty healthy lowlanders (15 males and 5 females) were recruited from a group of individuals planning to complete a 4 day ascent (Figure 1) in the Aostes Valley, Italy to a maximum altitude of 4554 m (Rifugio Capanna Regina Margherita, Italy) where they would spend two nights. Overnight physiological measurements (e.g., oxygen saturation and heart rate), clinical examinations and questionnaires were completed each day, as described in subsections below.

**FIGURE 1:**
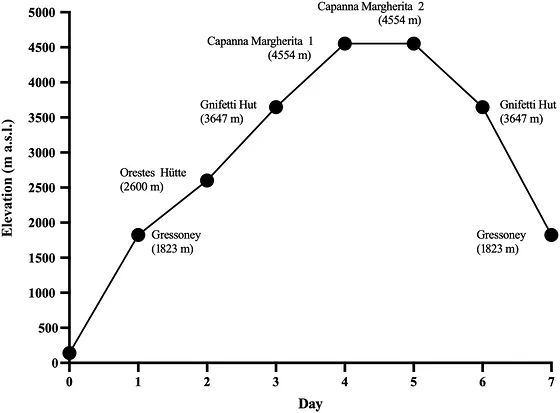
Ascent profile. Chairlifts and gondolas were used for parts of the ascent between Gressoney and Orestes Hütte and between Orestes Hütte and Gnifetti Hut.

### 2.3 Clinical evaluation and questionnaires

Lake Louise scores (LLS) were recorded each morning (Roach et al., 2018) using a bespoke smartphone application (H2 Cognitive Designs Ltd, Stoke‐on‐Trent, UK). Clinical evaluations were conducted by the senior study medical officer (Intensive Care and Anaesthetic Consultant Physician) each day. This included consultation, physical appearance and overall impression, with diagnosis of AMS being one of exclusion. The study medical officer also reviewed participants’ LLS (with the clinical function component) during the clinical evaluation. AMS classification (i.e., AMS vs. non‐AMS) was determined through careful review of the clinical presentation and LLS (AMS, total LLS ≥ 3 with headache ≥ 1; or non‐AMS, total LLS < 3 without headache). Individuals requiring treatment (e.g., immediate descent or oxygen or pharmacological therapy) for suspected altitude illness(es) were classified as having AMS regardless of LLS. The Groningen sleep quality questionnaire survey (GSQS) was completed each morning alongside the LLS using the bespoke smartphone application to assess sleep quality, with scores calculated in accordance with published criteria (Jafarian et al., 2008).

### 2.4 Overnight pulse oximetry

#### 2.4.1 Data collection

Peripheral oxygenation ($S_{pO_{2}}$) and heart rate (HR) were measured continuously (1 Hz) overnight during one night in the UK prior to departure (baseline/familiarisation) and each night during ascent using US Food and Drug Administration‐approved wrist‐worn pulse oximeters (WristOx_2_ 3150 Model, Nonin Medical Inc., Plymouth, MN, USA) and accompanying flexible finger sensors (PureLight® 8000J, Nonin Medical) with ±2% accuracy during movement and low perfusion (according to the manufacturer's claims). Oximeters were compliant with ISO 80601‐2‐61:2017, which sets the standard for basic safety and performance of medical equipment, and with ISO 80601‐2‐61:2019 guideline for accuracy testing for pulse oximeters (with ≤4% being acceptable accuracy) (Santos et al., 2022).

Oximeters were programmed to start recording once the finger sensor was inserted into the wrist‐worn component. Individuals were instructed to attach the oximeter (wrist‐worn) and finger sensor and to plug in the sensor as they lay down to sleep and remove it immediately upon waking, as previously described (K. E. Joyce et al., 2024).

#### 2.4.2 Preprocessing

Data were stored locally on device until offloaded using nVision (v.6.5.1, Nonin Medical) and saved to an ‘.asc’ text format for further processing (including artefact removal) using a bespoke MATLAB program. Artefacts were defined as: (1) $S_{pO_{2}}$ value <30%; (2) arbitrary $S_{pO_{2}}$ value >100% (e.g. 500% for WristOx_2_ devices); or (3) deviation in $S_{pO_{2}}$ of >4% or deviation in HR of ≥6 beats compared with the preceding measurement (Taha et al., 1997). The artefact index (AI), or the percentage of the total raw overnight recording identified as artefact, was estimated and reported to ensure the quality of recordings. A data cleaning algorithm was then applied such that both the $S_{pO_{2}}$ and HR data points were removed if an artefact was detected in either signal, and linear interpolation was applied per unit time to fill the instantaneous missing data points prior to analysis.

#### 2.4.3 Feature extraction and engineering

Following artefact detection, removal and cleaning, biomarkers were extracted from both the time and frequency domains (Gutiérrez‐Tobal et al., 2019) for overnight HR and $S_{pO_{2}}$ signals. Extracted biomarkers were determined by careful review of the literature for similar applications (Alvarez et al., 2010) and previous altitude studies (Cross et al., 2015) (Table 1), with description of and rationale for extracted biomarkers presented in the proceeding subsections.

**TABLE 1:** Oximetry‐based ($S_{pO_{2}}$ and HR signals) biomarkers extracted and considered for feature selection.

			Oximetry‐based biomarkers ($S_{pO_{2}}$ signal)			
		Abbreviation	Item	Definition	Units	Reference
	**Time domain**	**Common/descriptive statistics**				
1		*Mt1*	Mean $S_{pO_{2}}$	Arithmetic mean, central tendency	%	Alvarez et al. (2010)
2		Mt2	Variance of overnight $S_{pO_{2}}$	Describes the oscillation (squared deviations) around the mean	%^2^	Alvarez et al. (2010)
3		Mt3	Skewness of overnight $S_{pO_{2}}$	Describes the direction of the tail of the $S_{pO_{2}}$ histogram	nu	Alvarez et al. (2010)
4		Mt4	Kurtosis of overnight $S_{pO_{2}}$	Describes the peakedness of saturation from the $S_{pO_{2}}$ histogram	nu	Alvarez et al. (2010)
5		$S_{pO_{2}\min}$	Minimal $S_{pO_{2}}$	The absolute lowest instantaneous $S_{pO_{2}}$ measurement exhibited throughout the entire overnight recording	%	
6		$S_{pO_{2}\max}$	Maximal $S_{pO_{2}}$	The absolute highest instantaneous $S_{pO_{2}}$ measurement exhibited throughout the entire overnight recording		
7		SD $S_{pO_{2}}$	$S_{pO_{2}}$ standard deviation	Describes the square root of the oscillations around the average	%	
		**Conventional/complexity indices**				
8		$S_{pO_{2}}$‐CV	Coefficient of variation of $S_{pO_{2}}$			Tellez et al. (2016)
9		Δ1st–2nd	Change from first to second halves of the night	Defined as the difference between the means of the first and second half of overnight recordings normalised to the mean of the first half of the night (i.e., percentage increase/decrease relative to first half)	%	Tannheimer et al. (2017)
		**Desaturation measures**				
10		Ds	Total desaturation events	Desaturation event was defined as a drop in $S_{pO_{2}}$ by ≥4% (point B) from baseline (point A) that lasts ≥10 s and ≤60 s	events	Cross et al., (2015); Taha et al. (1997)
11		ODIx	Oxygen desaturation index	Described as the number of desaturation events that occur per unit time. This is estimated as the total number of desaturations during the recording normalised to recording length	events/h	Cross et al. (2015)
12		Lowest desat	Lowest desaturation	The lowest percentage drop in $S_{pO_{2}}$ from baseline across all desaturation events from the overnight recording	%	
		**Hypoxic burden**				
13		HB	hypoxic burden	HB is defined as the sum of the area under the individual saturation curves divided by total sleep/recording time	%min/h	Azarbarzin et al. (2019)
14		AODmax	Area under the desaturation curve	Area under the oxygen desaturation event curve, using the maximum $S_{pO_{2}}$ value as baseline (average taken over first 5–10 min used as baseline) and normalised by the total recording time	%	Kulkas et al. (2013); Levy, Álvarez, Rosenberg et al. (2021)
15		AOD_100_	Cumulative area under the desaturations curve	Cumulative area under the oxygen desaturation event curve, using the 100% $S_{pO_{2}}$ level as baseline and normalised by the total recording time	%	Levy, Álvarez, Rosenberg et al. (2021)
16		CTx (or TSTx)	Cumulative time spent be spent below x% $S_{pO_{2}}$	Cumulative time spent be spent below x% $S_{pO_{2}}$ normalised to the duration of overnight recording, with x evaluated for each $S_{pO_{2}}$ level between 50% and 100% $S_{pO_{2}}$ (also known as the total saturation time of x%, TST80)	%	Cross et al. (2015)
		**Non‐linear/complexity biomarkers**				
17		CTM	Central tendency measure	A non‐linear measure that assesses the degree of variability in physiological data with higher variability corresponding to lower CTM	nu	Cohen et al. (1996); Alvarez et al. (2007)
18		LZC	Lempel–Ziv complexity	LZC evaluates the degree of complexity of spatiotemporal patterns within the $S_{pO_{2}}$ signal	nu	Lempel & Ziv (1976)
19		SampEn	Sample entropy		nu	Richman & Moorman (2000)
20		ApEnt	Approximate entropy		nu	Pincus & Singer (1996); Richman & Moorman (2000)
	**Frequency domain**	**Whole‐spectrum analysis/non‐stationary**				
21		Mf1	Arithmetic mean, central tendency of frequency domain/power spectral density		dB	Alvarez et al. (2010)
22		Mf2	Variance of frequency domain	Variance of the PSD for the whole frequency domain	%^2^	Alvarez et al. (2010)
23		Mf3	Skewness of frequency domain	Skewness of frequency domain/spectral skewness of the PSD function for the whole frequency domain	nu	Alvarez et al. (2010)
24		Mf4	Kurtosis of frequency domain	Spectral kurtosis of the PSD function for the whole frequency domain	nu	Alvarez et al. (2010)
25		MF	Median frequency of frequency domain (i.e., PSD)	Frequency value at which 50% of all spectral power values from the $S_{pO_{2}}$ recording fall below	Hz	Alvarez et al. (2007)
26		SpecEn	Spectral entropy	SpecEn is a disorder quantifier related to the flatness of the spectral content	nu	
27		PA	Peak amplitude of PSD function	Corresponds to the peak amplitude of the PSD.	dB/Hz	
28		P_T_	Total power, also known as PSDtotal, absolute spectral power	Corresponds to the area defined by the power spectrum	dB	
		**Specific frequency band (0.014–0.033 Hz)**				
29		P_band_	Total power for the specified frequency band, also known as also known as PSD_band_	Total spectral power within the OSA band 0.014–0.033 Hz****Integral of PSD f(x) within the band 0.014–0.033 Hz (in OSA band; P_R_)	dB	Zamarrón et al. (1999)
30		P_R_	Relative power, also known as PSD_ratio_	Corresponds to the ratio between total power of a specific spectral band (PSDband; i.e., 0.017–0.033 Hz) and the total power of the signal (area; or P_T_ or PSDtotal; i.e., absolute spectral power)	dB	Zamarrón et al. (1999)
31		PA_x_	Peak amplitude of PSD function within ‘x’ frequency band, also known as PSDpeak	Corresponds to the peak amplitude of the PSD function within the 0.014–0.033 Hz frequency band	dB/Hz	Terrill (2019); Zamarrón et al. (1999)
32		PF	Peak frequency	Peak frequency is the frequency at which the PSD has the highest magnitude (i.e., at the peak amplitude) for the OSA band	Hz	
33		*Mf1*_band_	Average power of the PSD function within the specified ‘band’		dB	Terrill (2019)
34		*Mf2*_band_	Variance of the power spectral density function within the specified ‘band’	Describes the oscillation around the mean for the PSD	%^2^	Alvarez et al. (2010)
35		*Mf3*_band_	Spectral kurtosis of the PSD function within the specified ‘band’	Describes the peakedness of the PSD histogram within the band of interest	nu	Alvarez et al. (2010)
36		*Mf4*_band_	Spectral skew of the PSD function within the specified ‘band’	Describes the direction of the tail of the PSD histogram within the band of interest	nu	Alvarez et al. (2010)
			**Oximetry‐based biomarkers (HR signal)**			
37		Mt1_HR_	Mean HR	Arithmetic average of heart rate over the entire recording	beats/min	
38		SD_HR_	SD HR	Standard deviation of HR across the entire recording	(beats/min)^2^	
39		HR_min_	Minimum HR	Lowest or nadir (instantaneous) HR observed during the recording	beats/min	
40		HR_max_	Maximum HR	Highest (instantaneous) HR observed during the recording	beats/min	
41		HR/$S_{pO_{2}}$	Heart rate to $S_{pO_{2}}$ ratio	A measure of balance within the cardiopulmonary system in response to hypoxic stimulus.	beats/min/%	K. Joyce et al. (2024)
42		HR events	Number HR events	No. of events classified as a rise in heart rate by six or more beats from preceding baseline which lasts for ≥8 s	no. of events	
43		HR events/h		No. of total HR events normalised to the length of the entire recording and presented as the average number of events per hour	events/h	

*Note*: Oximetry biomarkers to be extracted from the $S_{pO_{2}}$ and HR signals, which have been collated from biomarkers presented by Levy, Álvarez, Rosenberg et al. (2021) and Levy, Álvarez, Del Campo et al.  2021, Alvarez et al. (2010) and Terrill (2019). Also included are non‐oximetry biomarkers. Abbreviations and units of measure are all presented for all units.

Metrics related to HRV may only be attainable from Garmin devices and will therefore be unlikely to be included in the predictive modelling related to oximetry measurements obtained from the Nonin finger oximeter (3150 Model WristOx_2_, Nonin Medical).

#### 2.4.4 Time domain

##### 2.4.4.1 Common/descriptive statistics

Common descriptive statistics were extracted from overnight $S_{pO_{2}}$ and HR data for each overnight recording as previously described (Alvarez et al., 2010) and included the minimum, maximum, arithmetic mean ± SD (first‐order statistical moment, *Mt1*) and median (25%–75% interquartile range). The arithmetic mean of the HR/$S_{pO_{2}}$ ratio was also estimated for each recording and provides a measure of cardiopulmonary‐linked responses (in the absence of HR variability data) (Botek et al., 2015). Likewise, the second‐ to fourth‐order statistical moments [i.e., variance (*Mt2*), skewness (*Mt3*) and kurtosis (*Mt4*)] were extracted from the relative frequency plots of the $S_{pO_{2}}$ time series.

##### 2.4.4.2 Conventional indices

In addition to the common descriptive statistics, conventional $S_{pO_{2}}$ and HR events were detected and characterised. The $S_{pO_{2}}$ events were defined as a minimum drop in $S_{pO_{2}}$ by 4% (or rate of 0.1%/s) from a preceding 20 s baseline, lasting ≥10 s (Taha et al., 1997). These events were counted across the nocturnal recording (i.e., total $S_{pO_{2}}$ events) and also expressed as an index per unit time [i.e., oxygen desaturation index (ODI_4%_), desats/h]. Likewise, HR events were defined as an increase in HR by ≥6 beats/min with the change lasting ≥8 s. To understand the overnight hypoxic insult, cumulative time (CT) or total saturation time (TST) spent below each $S_{pO_{2}}$ between 0% and 100% $S_{pO_{2}}$ (e.g., TST < 80%; Cross et al., 2015) and hypoxic burden [%min/h or desaturation area (Azarbarzin et al., 2019; Pimenta et al., 2010); or percentage desaturation] were extracted. Less common ‘conventional’ indices that were also extracted, including: (1) the change in $S_{pO_{2}}$ from the first to the second half of the night, given its potential relevance for the detection of AMS (Tannheimer et al., 2017); and (2) coefficient of variation of overnight $S_{pO_{2}}$ ($S_{pO_{2}}$‐CV), which has been considered an indirect measurement of altered ventilatory drive (Bettinardi, 2009; Tellez et al., 2016).

##### 2.4.4.3 Non‐linear biomarkers

Lastly from the time domain, several non‐linear biomarkers were extracted, including: central tendency measure (CTM; Cohen et al., 1996; Alvarez et al., 2007), Lempel–Ziv complexity (LZC; Lempel & Ziv, 1976), sample entropy (SampEn) and approximate entropy (ApEnt; Pincus & Singer, 1996; Richman & Moorman, 2000). LZC, a non‐parametric measure of complexity, required the $S_{pO_{2}}$ signal initially to be converted to a binary sequence by comparing each measurement with a predefined threshold (in this case, the average). A complexity counter was then used to count each new subsequence within the binary sequence, *c*(*n*). These counts were then divided by a normalisation parameter *b*(*n*) as previously described (Álvarez et al., 2012).

#### 2.4.5 Frequency domain

##### 2.4.5.1 Whole‐spectrum analysis

From the frequency domain, the power spectral density (PSD) of the $S_{pO_{2}}$ signals was estimated (across the entire power spectrum) using Welch's non‐parametric periodogram (Gutiérrez‐Tobal et al., 2019; Welch, 1967). This method divides the signal into ‘*n*’ overlapping segments of a designated length (*l*) and computes a periodogram for each windowed segment using the specified discrete Fournier transform. The amplitude of the PSD at each spectral component was used to obtain the normalised histogram for the frequency domain. From this, the first to fourth (*Mf1–Mf4*)‐order statistical moments were estimated for the frequency domain and included: mean (*Mf1*), variance (*Mf2*), skewness (*Mf3*) and kurtosis (*Mf4*). The median frequency (MF), or the Frequency value at which 50% of all spectral power values from the SpO_2_ recording fall below (Alvarez et al., 2007), was also extracted from the frequency domain, along with the estimate of spectral entropy (SpecEnt).

##### 2.4.5.2 Specific frequency band analysis

First to fourth (*Mf1–Mf4*)‐order statistical moments (i.e., mean, variance, skewness and kurtosis) were also extracted from the isolated 0.014–0.033 Hz frequency band, which has proved to be sensitive for the detection of sleep‐disordered breathing (SDB; e.g., obstructive sleep apnoea; Alvarez et al., 2010; Sutherland et al., 2022). Additional biomarkers were extracted exclusively from the 0.014–0.033 Hz frequency band and included: peak amplitude (PA, and corresponding frequency), total power (P_T_; total spectral power) and relative power (P_R_; function/ratio of total power exhibited within the obstructive sleep apnoea (OSA) band relative to the total spectral power overall; Alvarez et al., 2010).

### 2.5 Statistical analysis

#### 2.5.1 Univariate analysis

Biomarkers for the $S_{pO_{2}}$ and HR signals are presented as the median ± (25%–75%) interquartile range. Mixed‐effects analysis (or ANOVA) with Sidak's *post hoc* correction was used to compare between AMS and non‐AMS subgroups (as categorised by clinical examinations) across days of ascent for all biomarkers (from both $S_{pO_{2}}$ and HR signals). All statistical analyses were performed using a combination of MATLAB and Prism (v.9.4.0, Graphpad Software LLC, San Diego, CA, USA). All statistical tests were two‐tailed, with significance set to α < 0.05, with adjusted *P*‐values reported.

#### 2.5.2 Exploratory multivariate analysis

Data were grouped based on the night of collection, with feature selection and model training/validation performed separately for each night of ascent (3 nights in total), as described in Appendix 1. Nightly models were then evaluated based on model performance metrics (e.g., accuracy), as also described in Appendix 1.

## 3 RESULTS

All twenty individuals (15males and 5 females, 36.8 ± 18.5 years old) ascended successfully to 4554 m.

### 3.1 Clinical examinations and questionnaires

Across both mornings at 4554 m, 11 individuals were identified as having AMS on clinical examination, and 15 were identified from LLS. Of the 11 clinically identified cases, LLS correctly identified 10 of 11 cases.

Results for daily GSQS scores are presented in Figure 2a. Despite the higher mean GSQS scores among AMS individuals at Orestes (2600 m), Gnifetti (3647 m) and Margherita Huts (4554 m), no significant effect of AMS status was observed (*P =* 0.085), nor was any interaction effect (AMS status × time/altitude) observed (*P =* 0.111). Exploratory analysis demonstrated a significant relationship between GSQS and LLS (Figure 2b).

**FIGURE 2:**
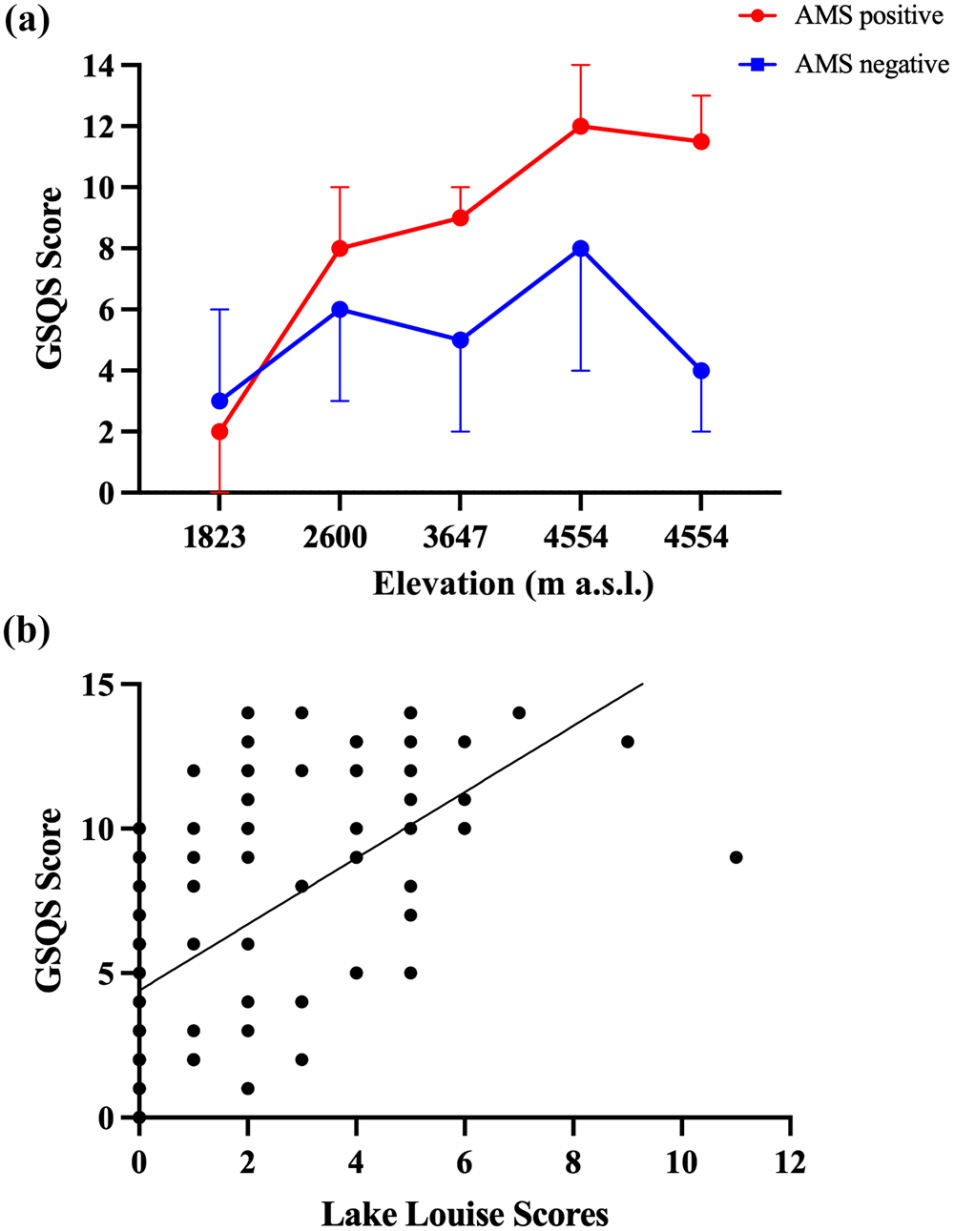
(a) Daily GSQS scores during ascent. Data are plotted as the median ± interquartile range (25%–75%). Results from mixed‐effects analysis with Sidak's correction demonstrated no significant differences between AMS and non‐AMS subgroups at any of the time points throughout ascent for GSQS scores. A significant effect of altitude (*P <* 0.001) was observed for GSQS. (b) Results from exploratory analysis for simple linear regression between GSQS and Lake Louise scores. Exploratory analysis demonstrated a significant (albeit weak) relationship between GSQS and Lake Louise scores (*r* ^2^ = 0.357, *P <* 0.001). Abbreviations: AMS, acute mountain sickness; GSQS, Groningen sleep quality scale.

### 3.2 Pulse oximetry recordings

Ninety‐three of 100 oximetry recordings (including recordings from 4554 m) were obtained successfully, with a total of 743.1 h (average recording length, 8.0 ± 1.0 h) of data collected, and limited artefacts observed (1.1% ± 1.3%).

#### 3.2.1 Univariate analysis

Biomarkers were divided by subgroups (AMS vs. non‐AMS, as determined from clinical examinations at 4554 m) and are presented as the median ± interquartile range in Figures 3, 4, 5, 6, 7. Time series biomarkers (from both $S_{pO_{2}}$ and HR signals) are presented in Figure 3. Desaturation characteristics and HR event metrics are presented in Figure 4, with additional conventional indices related to desaturation time presented in Figure 5. Biomarkers extracted from the frequency domain are presented in Figures 6 and 7 (specific to the 0.014–0.033 Hz frequency band). Finally, non‐linear/complexity biomarkers are presented in Figure 8.

**FIGURE 3:**
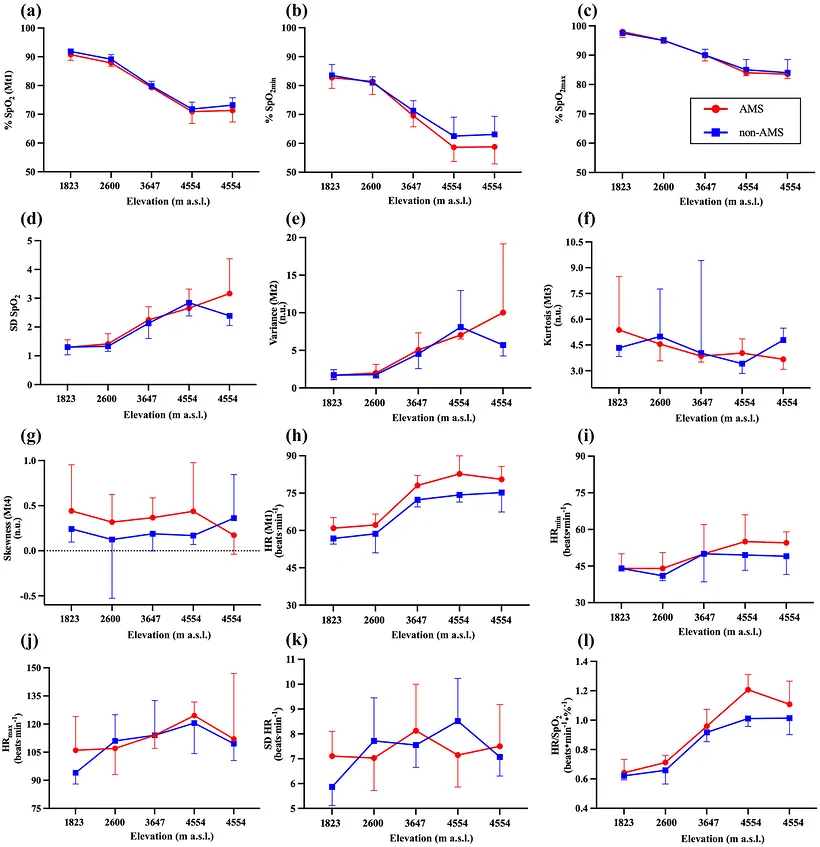
Time series metrics extracted from overnight pulse oximetry recordings during ascent. Daily values during ascent for: (a) Mean of peripheral oxygenation ($S_{pO_{2}}$ (Mt1)); (b) peripheral oxygenation minimum ($\%S_{pO_{2}\min}$); (c) peripheral oxygenation maximum ($\%S_{pO_{2}\max}$); (d) standard deviation of $S_{pO_{2}}$ overnight (SD $S_{pO_{2}}$); (e) $S_{pO_{2}}$ variance (Mt2); (f) $S_{pO_{2}}$ kurtosis (Mt3); (g) $S_{pO_{2}}$ skewness (Mt4); (h) mean heart rate (HR (Mt1)); (i) heart rate minimum (HRmin); (j) heart rate maximum (HRmax); (k) standard deviation of heart rate (SD HR); (l) ratio of heart rate to peripheral oxygenation (HR/$S_{pO_{2}}$). Metrics are presented for data extracted from both $S_{pO_{2}}$ and HR signals. *Mt1* (mean), *Mt2* (variance), *Mt3* (kurtosis) and *Mt4* (skewness) refer to the statistical moments of the time series data for $S_{pO_{2}}$ recordings. Data correspond to mixed‐effects analysis results presented in Table 2. A significant main effect of time/altitude was observed for $S_{pO_{2}\min}$, $S_{pO_{2}\max}$, *Mt1*_SpO2_, variance (*Mt2*), SD_SpO2_, *Mt1*_HR_, HR_min_, SD_HR_ and HR/$S_{pO_{2}}$ (all *P <* 0.01). Only HR/$S_{pO_{2}}$ also showed a significant main effect for the AMS subgroup (*P =* 0.020). No effect of interaction (time/altitude × AMS subgroup) was observed. Abbreviations: AMS, acute mountain sickness; HR, heart rate; $S_{pO_{2}}$, peripheral oxygenation.

**FIGURE 4:**
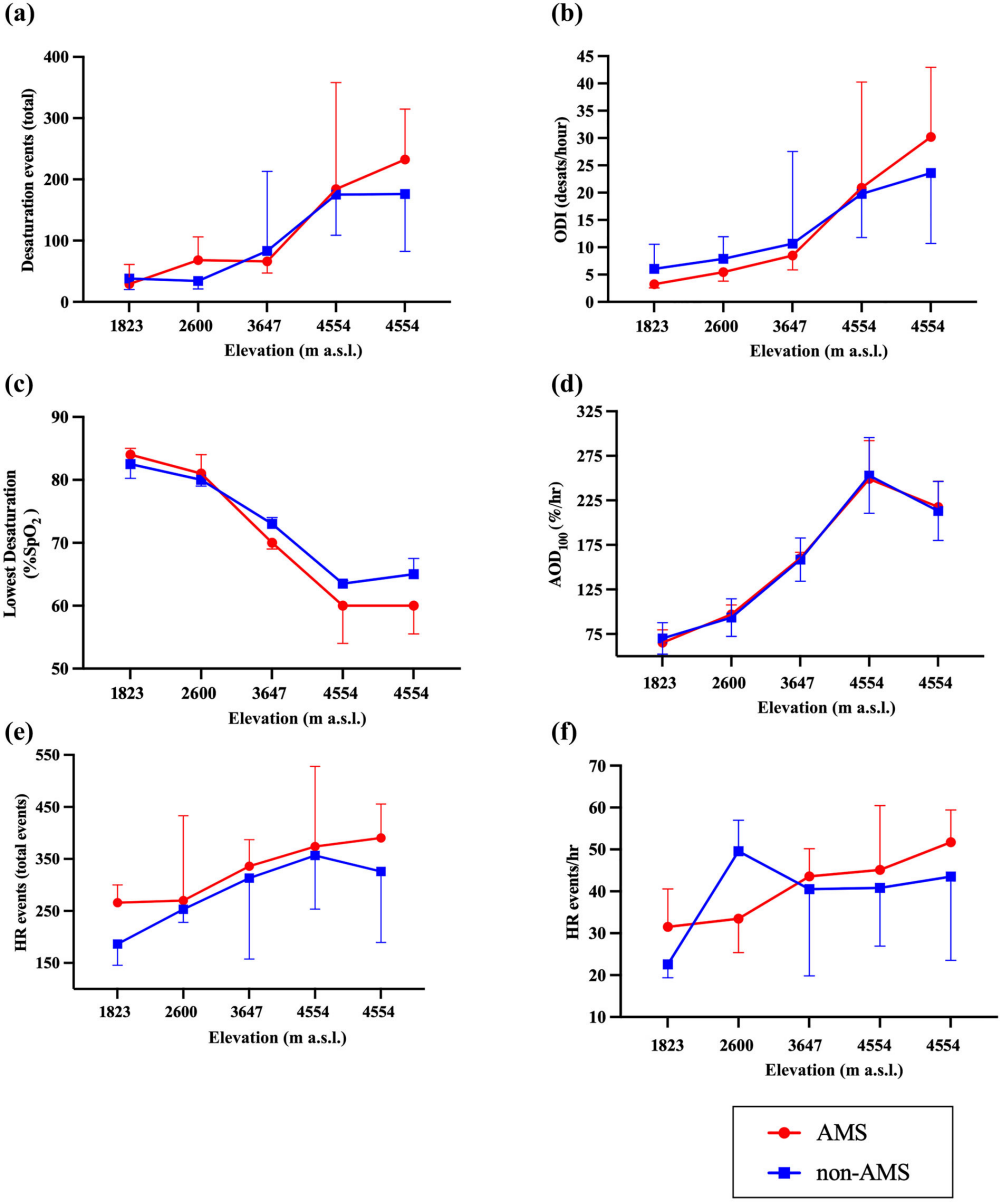
Desaturation characteristics and heart rate events. (a) Desaturation events from peripheral oxygenation. Events were defined as a minimum drop in $S_{pO_{2}}$ by 4% (or rate of 0.1%/sec) from a preceding 20 second baseline, and lasting at least 10 seconds (Taha et al., 1997). (b) Oxygen desaturation index. events were counted across the nocturnal recording (i.e., total $S_{pO_{2}}$ events) and further expressed as an index per unit time (i.e., oxygen desaturation index (ODI4%), desats/hour). (c) Lowest desaturation ($\%S_{pO_{2}}$) reflects the most severe desaturation (lowest $S_{pO_{2}}$ during a desaturation event) exhibited overnight. (d) Area of desaturation. (e) Heart rate events reflect the number of events over the course of the night with HR events defined as drop in HR by at least 6 bpm with the change lasting at least 8 seconds. (f) Heart rate events per hour reflect the number of HR events per unit time over the course of the overnight recording. Nightly data are plotted as the median ± interquartile range separately for subgroups (AMS vs. non‐AMS) and correspond to mixed‐effects comparisons reported in Table 2, with significance set to α < 0.05. A significant main effect of time/altitude was observed for desaturation events (total), ODI, HR events (total), HR events/h and AOD_100_ (all *P <* 0.01). No significant effect was observed for AMS subgroup or interaction. Abbreviations: AMS, acute mountain sickness; AOD_100_, cumulative area under the desaturations curve; HR, heart rate; ODI, oxygen desaturation index.

**FIGURE 5:**
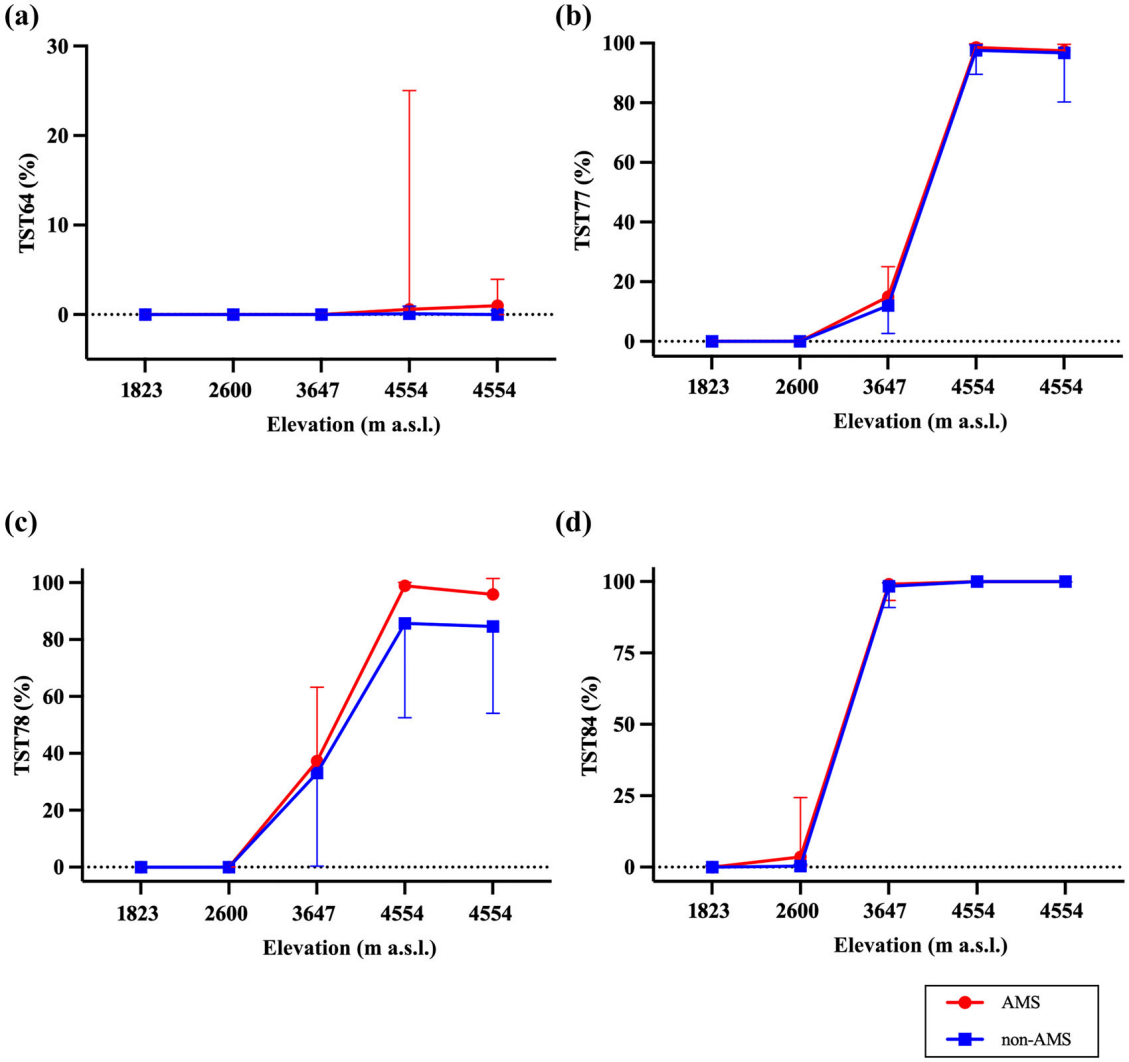
Conventional indices for total saturatoin time (TSTx) spent at or below specific peripheral oxygenation levels. (a) Total saturation time spent below 64% $S_{pO_{2}}$ (TST64). (b) Total saturation time spent below 77% $S_{pO_{2}}$ (TST77). (c) Total saturation time spent below 78% $S_{pO_{2}}$ (TST78). (d) Total saturation time spent below 84% $S_{pO_{2}}$ (TST84). Nightly data are plotted as the median ± interquartile range separately for subgroups (AMS vs. non‐AMS) except (a), which is plotted as mean ± SEM. Plotted data correspond to mixed‐effects analysis reported in Table 2, with significance set to α < 0.05. A significant main effect of time/altitude was observed for TST77, TST78 and TST84 (all *P* < 0.01). Total saturation time (TSTx) or cumulative time (CTx) spent at or below a specific saturation level.

**FIGURE 6:**
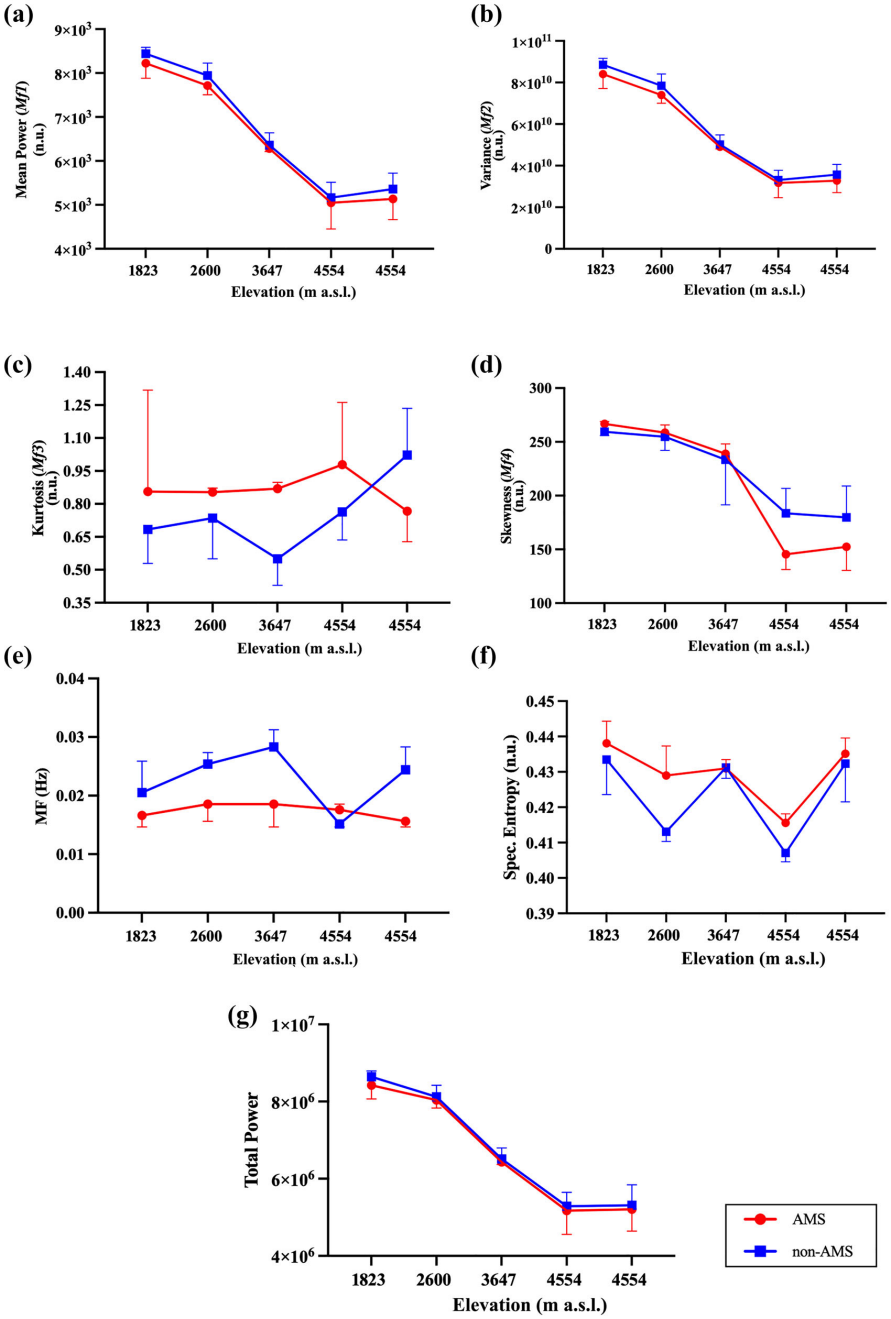
Nightly data for metrics related to power spectral density and extracted from the frequency domain. Nightly data are plotted as the median ± interquartile range separately for subgroups (AMS vs. non‐AMS). Statistical moments for the frequency domain are labelled as: (a) *Mf1* (mean), (b) *Mf2* (variance), (c) *Mf3* (kurtosis) and (d) *Mf4* (skewness). Additional measures extracted from the freuqnecy domain are also presented: (e) Median frequency, (f) Spectral entropy, and (g) total power. Plotted data correspond to mixed‐effects analysis reported in Table 2, with significance set to α < 0.05. A significant main of time/altitude was observed for *Mf1*, *Mf2*, *Mf4*, MF, total power and spectral entropy (all *P <* 0.05). Abbreviations: AMS, acute mountain sickness; MF, median frequency; spec. entropy, spectral entropy.

**FIGURE 7:**
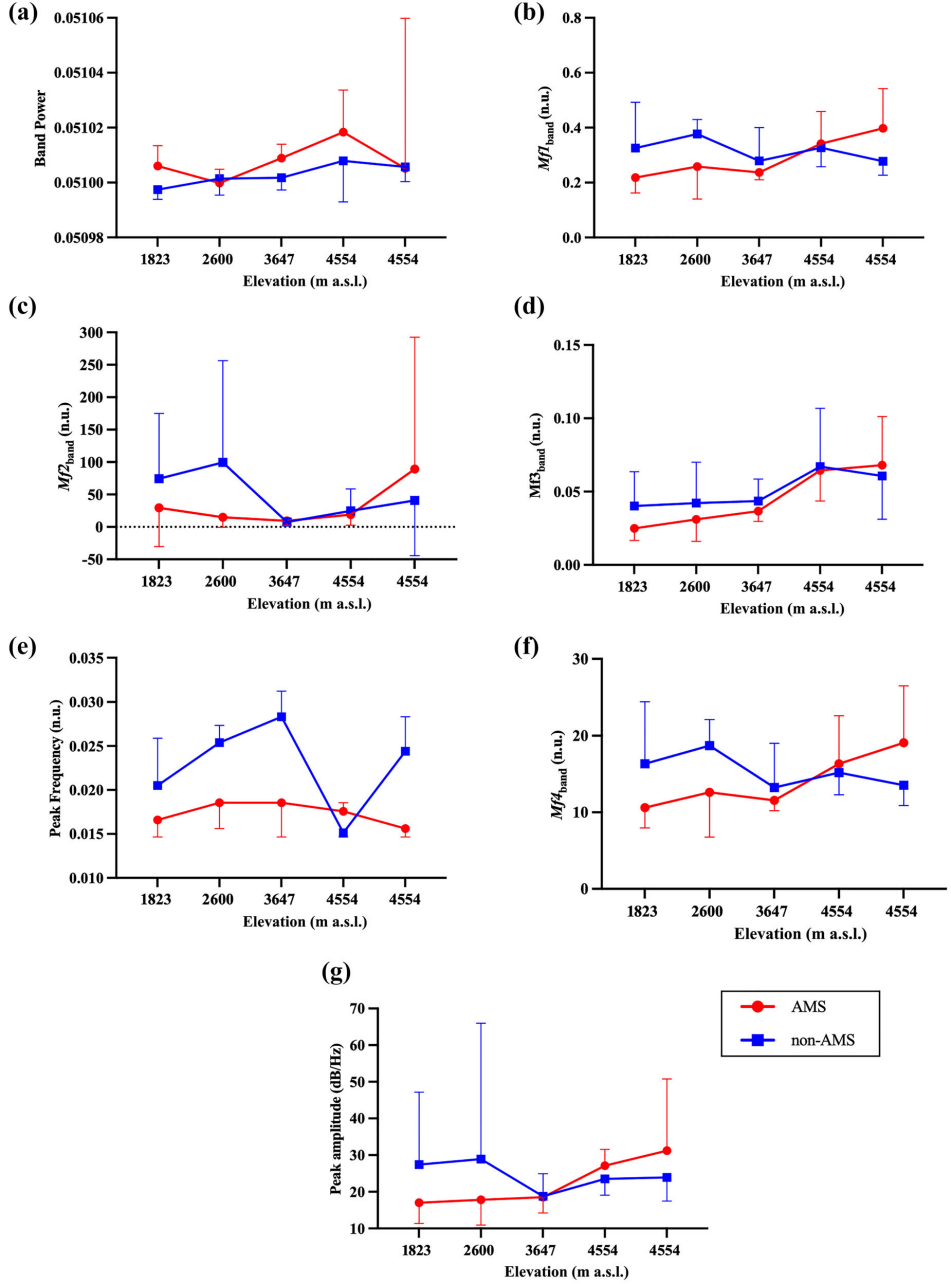
Power spectral density metrics extracted from the frequency domain. Statistical moments are presented metrics specific to the 0.014–0.033 Hz frequency band and include: (b) mean (*Mf1*_band_), (c) variance (*Mf2*_band_), (d) kurtosis (*Mf3*_band_) and (f) skewness (*Mf4*_band_). Additional power spectral density measures from overnight recordings are also presented: (a) band power, (e) peak frequency and (g) peak amplitude. Nightly data are plotted as the median ± interquartile range separately for subgroups ( AMS vs non‐AMS). Plotted data correspond to mixed‐effects analysis results presented in Table 2, with significance set to α < 0.05. A significant main effect of time/altitude was observed for *Mf3*_band_ and peak frequency (both *P <* 0.05). Abbreviation: AMS, acute mountain sickness.

**FIGURE 8:**
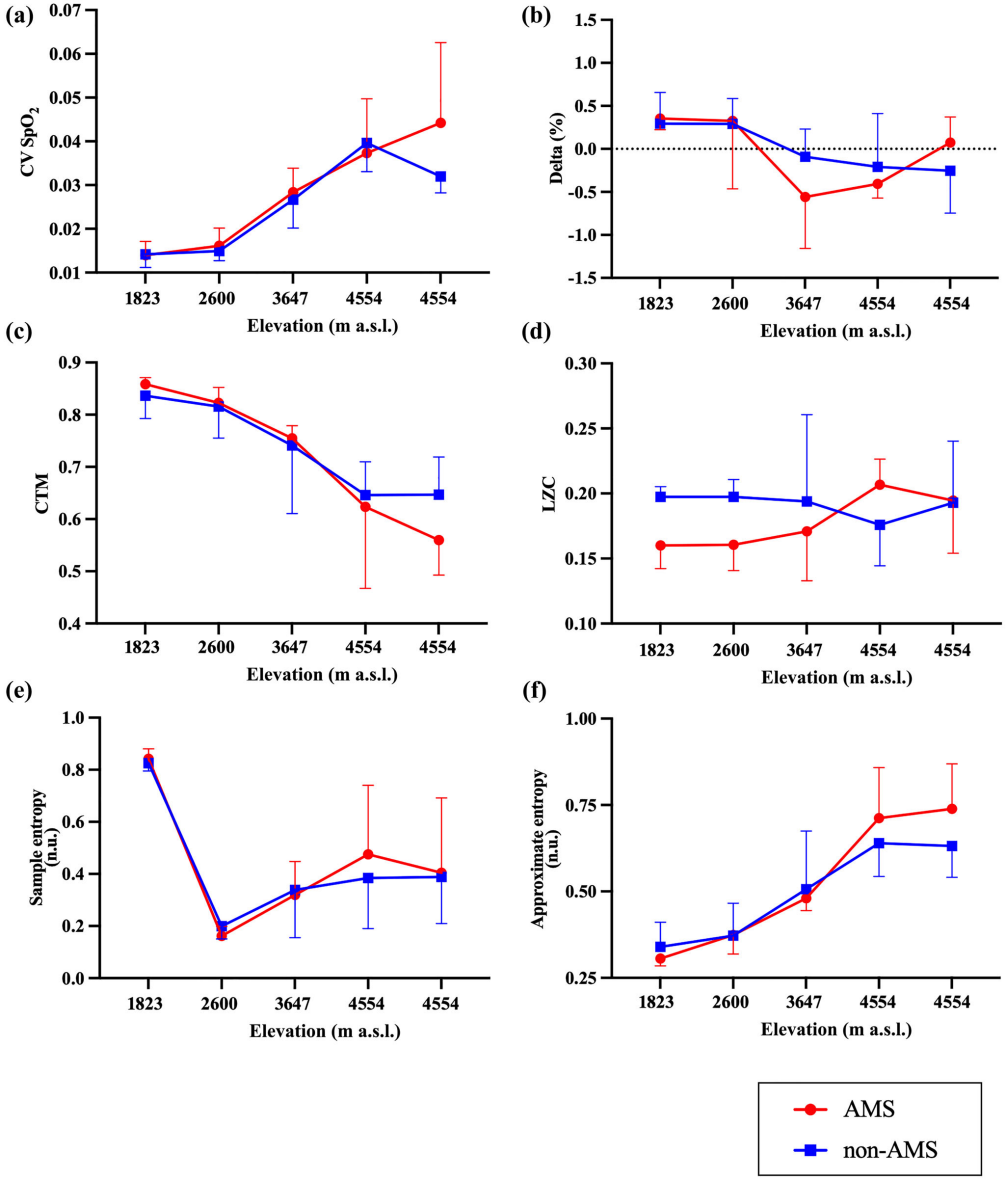
Non‐linear/complexity features:(a) Coefficient of variation (CV) of $S_{pO_{2}}$; (b) Delta (%) or change in $S_{pO_{2}}$ from first to second halves of the night; (c) central tendency measure (CTM); (d) Lempel‐Ziv complexity (LZC); (e) sample entropy; (f) approximate entropy. Overnight biomarkers extracted from oximetry recordings are plotted as the median ± interquartile range separately for subgroups (AMS vs. non‐AMS). Plotted data correspond to mixed‐effects analysis reported in Table 2, with significance set to α < 0.05. A significant main effect of time/altitude was observed for CTM sample entropy and approximate entropy (all *P <* 0.01).

Results from mixed‐effects analysis for comparisons between AMS and non‐AMS subgroups for oximetry biomarkers across ascent are presented in Table 2. To avoid redundancy, only TSTx measures that were subsequently selected during feature selection processes for classification models were included in Table 2.

**TABLE 2:** Results from mixed‐effects analysis (with Sidak's correction) comparing AMS vs. non‐AMS (AMS status) across days of ascent (i.e., time/altitude) for overnight oximetry biomarkers ($S_{pO_{2}}$ and HR signals).

Feature	Time (or altitude)	AMS status	Interaction effect
**Descriptive statistics and statistical moments**			
$S_{pO_{2}\min}$	<0.001^*^	0.194	0.293
$S_{pO_{2}\max}$	<0.001^*^	0.885	0.845
*Mt1*_SpO2_ (mean)	<0.001^*^	0.255	0.255
SD_SpO2_	<0.001^*^	0.300	0.503
*Mt2*_SpO2_ (variance)	<0.001^*^	0.278	0.302
*Mt3*_SpO2_ (kurtosis)	0.213	0.682	0.078
*Mt4*_SpO2_ (skewness)	0.454	0.734	0.501
HR_min_	0.001^*^	0.117	0.481
HR_max_	0.242	0.461	0.374
*Mt1*_HR_	<0.001^*^	0.070	0.410
SD_HR_	0.009^*^	0.798	0.324
HR/$S_{pO_{2}}$	<0.001^*^	0.020^*^	0.137
**Conventional/complexity indices**			
$S_{pO_{2}}$‐CV	<0.001^*^	0.274	0.452
first2sec	0.003^*^	0.878	0.057
**Non‐linear/complexity biomarkers**			
CTM	<0.001^*^	0.923	0.239
LZC	0.125	0.311	0.236
SampEn	<0.001^*^	0.888	0.799
ApEnt	<0.001^*^	0.991	0.487
**Desaturations and HR events**			
Ds	<0.001^*^	0.805	0.742
ODIx	<0.001^*^	0.925	0.375
Lowest desaturation	<0.001^*^	0.177	0.174
HR events	0.001^*^	0.482	0.923
HR events/h	0.001^*^	0.578	0.200
**Hypoxic burden**			
AOD_100_	<0.001^*^	0.764	0.481
TST64	0.082	0.550	0.832
TST77	<0.001^*^	0.556	0.467
TST78	<0.001^*^	0.319	0.664
TST84	<0.001^*^	0.385	0.720
**Whole‐spectrum analysis/non‐stationary**			
*Mf1*	<0.001^*^	0.281	0.288
*Mf2* (variance)	<0.001^*^	0.371	0.477
*Mf3* (kurtosis)	0.388	0.250	0.776
*Mf4* (skewness)	<0.001^*^	0.483	0.168
MF	0.010^*^	0.093	0.408
SpecEn	<0.001^*^	0.052	0.476
P_T_	<0.001^*^	0.312	0.289
**Specific frequency band (0.014–0.033 Hz)**			
P_band_	<0.001^*^	0.312	0.289
P_R_	0.091	0.374	0.571
PA	0.151	0.532	0.121
PF	0.010^*^	0.093	0.408
*Mf1*_band_ (mean)	0.108	0.781	0.108
*Mf2*_band_ (variance)	0.239	0.458	0.235
*Mf3*_band_ (kurtosis)	0.029^*^	0.780	0.170
*Mf4*_band_ (skewness)	0.139	0.767	0.110

*Note*: Significance was set to α < 0.05, with significant results (adjusted *P*‐values presented) marked by an asterisk (^*^).

Abbreviations: AMS, acute mountain sickness; MF, median frequency; PA, peak amplitude; P_band_, total power in specific band (i.e., 0.014–0.03 Hz); PF, peak frequency; P_R_, relative power; P_T_, total power.

#### 3.2.2 Exploratory multivariate analysis

Results for the two‐step approach to feature selection are presented alongside optimised classification models in Appendix 2. Performances of classification models developed from each of the different feature selection methods for overnight biomarkers leading up to Margherita Hut (4554 m) are also presented in Appendix 2. The KNN classification model using LZC, median frequency and HR_min_ (from Orestes Hütte, 2600 m), selected via the Kruskal–Wallis *H* feature selection method, exhibited the highest accuracy (100%) for predictions of clinical AMS at 4554 m. Predictions from this model (for Orestes Hütte data) are plotted against two of these selected biomarkers (MF × LZC) and compared with the same biomarkers from Gnifetti Hut for visualisation purposes in Appendix 3. Exploratory analysis of model performances with fewer selected biomarkers (i.e., one or two instead of three biomarkers) did not show any improvements with fewer biomarkers selected across selection methods.

## 4 DISCUSSION

The purpose of this pilot study was to measure overnight oximetry during ascent to high altitude, extract biomarkers from overnight recordings and compare biomarkers between AMS and non‐AMS trekkers. Further exploratory work was conducted to assess whether biomarkers were predictive of AMS using preliminary classification models, with this aspect meant to serve largely as proof of concept for a larger‐scale, high‐altitude study.

To our knowledge, this is the first civilian study to extract and evaluate the range of oximetry biomarkers outlined herein from overnight oximetry recordings collected during ascent to high altitude and compare these between AMS and non‐AMS subgroups. Although there are other predictive models (Beidleman et al., 2013; Li et al., 2025; Zhang et al., 2026), this appears to be the first preliminary application of machine learning models to these oximetry biomarkers for the purpose of predicting AMS occurring days later, higher up the mountain.

We demonstrated that: (1) time, frequency and non‐linear biomarkers from overnight oximetry recordings exhibit different trends when compared between AMS and non‐AMS; and (2) overnight oximetry recordings might be useful for future predictive models for occurrence of AMS.

### 4.1 Univariate analysis

Although there were no significant differences between AMS and non‐AMS subgroups at any of the time points (from mixed‐effects analysis), it was clear that certain physiologically related trends occurred with ascent that were different between subgroups. These trends were observed in the time and frequency domains and for non‐linear biomarkers, as discussed below.

#### 4.1.1 Time series

With increasing altitude, most individuals exhibit an increase in SDB; however, the degree to which such SDB is related to AMS is unclear (Anderson et al., 2015). In the present study, the generalised increase in $S_{pO_{2}}$ variance (*Mt2*) was indicative of increased SDB with ascent for both subgroups. What was most interesting, however, was the dissociation between subgroups for these ascent‐related changes at the time of peak AMS symptoms (highest prevalence). More specifically, variance (*Mt2*) continues to rise at 4554 m in AMS, with opposite changes observed in non‐AMS. This morphology is also consistent with the generalised increase in oxygen desaturation index among both subgroups until 4554 m, at which point AMS individuals exhibit a further sharp increase (Figure 4b). This is also presented in the context of seemingly comparable $S_{pO_{2}}$ (*Mt1*) values throughout ascent (see Figure 3a). Individuals with AMS also exhibited a further decline in kurtosis and skewness with increasing altitude (Figure 3g,h). Taken together, results from statistical moments suggest a steadier and more concentrated $S_{pO_{2}}$ in non‐AMS, with a potential attenuation of SDB with acclimatisation (i.e., with back‐to‐back nights at 4554 m). Also, of note from the time series plots was the smaller change, on average, from the first to second half of the night for $S_{pO_{2}}$ among individuals with AMS compared with non‐AMS (Figure 8b), which was consistent with previous findings (Tannheimer et al., 2017).

#### 4.1.2 Frequency domain

PSD describes the frequency content of the oximetry signal, with the total power of a signal being equal to the sum of the absolute squares of its time‐domain values divided by the signal length, or total recording time in this case. Thus, with increasing altitude and the generalised decrease in $S_{pO_{2}}$ among all individuals (with and without AMS), it is no surprise that the total power decreased for both subgroups in a similar fashion.

Second to the total power, one can look at the changes in relative power (or total power within a specific frequency band) to evaluate the signal. For example, individuals with little to no SDB exhibit a relatively ‘non‐varying’ signal, with power concentrated at frequencies approaching zero (Terrill, 2019), whereas individuals with cyclical events exhibit frequency content associated with repetitive desaturations, which for OSA has been shown in the 30–70 s (or 0.014–0.03 Hz) band (Lin et al., 2009; Zamarrón et al., 1999). Thus, the increase in relative power (i.e., power in the specific frequency band) was unsurprising in light of the increase in sleep‐disordered breathing known to occur with ascent (Salvaggio et al., 1998). What was interesting, however, was that this rise in relative power was, on average, greater (at Orestes and Gnifetti Huts) and the peak amplitude higher (from 3647 m upwards) in individuals who went on to develop AMS at 4554 m (Figure 7g), notwithstanding that relative power or PA was not selected for the classification model. Likewise, the frequency of the dominant peak (i.e., peak frequency) was consistently lower on average in the AMS subgroup (see Figure 7e) compared with the non‐AMS subgroup, suggesting a longer desaturation period for AMS, which has previously been linked to pathophysiological sleep patterns (Terrill, 2019).

Taken together, this suggests that a certain degree of increase in SDB is likely to be physiological and essential for acclimatisation; however, at some threshold (i.e., around the time when dissociations/oppositional changes appear between AMS and non‐AMS) the disadvantages of high‐altitude periodic breathing begin to predominate and outweigh the benefits of attempts at increasing $S_{pO_{2}}$ (Küpper et al., 2008). This is supported by the drop/recovery in relative power with acclimatisation (i.e., 4554 m night 1 vs. night 2); however, further studies are required to confirm these findings. Additional analyses are also required to determine whether there is an alternative frequency band that is more indicative of adverse responses in $S_{pO_{2}}$ (or HR) overnight during ascent.

#### 4.1.3 Non‐linear/complexity biomarkers

Additional trends were evident in the non‐linear/complexity measures for AMS and non‐AMS. For example, LZC was on average higher in the non‐AMS subgroup up to 3647 m, above which these individuals exhibited a drop in LZC (at 4554 m), whereas individuals with AMS continued to exhibit a rise in LZC (as shown in Figure 8). Likewise, a lower CTM indicates a higher degree of chaos, which was demonstrated for individuals with AMS at 4554 m, and is indicative of greater variability (Marcos et al., 2009). This too might be indicative of the onset of changes in periodic breathing and the subsequent impact on $S_{pO_{2}}$ that occur with ascent to altitude (Salvaggio et al., 1998). Interestingly for non‐linear biomarkers, LZC was one of the biomarkers that was identified using the systematic approach to feature selection for optimal classification model development/testing and validation (refer to Table 2, discussed next). This is consistent with Álvarez et al. (2020), who also found LZC to be an important non‐linear feature to include when optimising predictive models, specifically in relationship to levels of hypoxia as they relate to adverse outcomes.

### 4.2 Exploratory multivariate analysis

The foundations of various methods used in this study were rooted in methodologies previously described for the detection of obstructive (and in some cases central) sleep apnoea from overnight pulse oximetry recordings (Gutiérrez‐Tobal et al., 2019). However, the basis for this study assumes that the morphology of overnight saturation (e.g., SDB/repetitive desaturations known to occur during ascent) is related to the occurrence/development of AMS during ascent. Unlike OSA studies, we did not expect to observe the same classification result for each overnight data set, but we expected that there is an altitude threshold during ascent that would permit the most favourable prediction of impending AMS occurring days later.

Evident in Appendix 2, different feature selection methods led to the selection of different biomarkers, which subsequently resulted in variable model performances. Although the same biomarkers might have been selected by multiple feature selection methods for the same overnight data set, only $S_{pO_{2}}$, approximate entropy and peak frequency were selected by multiple methods across multiple days/altitudes. Median frequency was selected across multiple days, albeit by the same selection method. The inconsistency of the selected biomarkers between selection methods and, particularly, between days of ascent is indicative of the changing morphology of overnight $S_{pO_{2}}$ that occurs with ascent and emphasises the importance of analysing the overnight data sets separately. The inconsistencies also reinforce the preliminary nature of this exploratory analysis. Kruskal–Wallis *H* and χ^2^ feature selection methods tended to select biomarkers that produced models with highest performances (see Appendix 2) and were considered most appropriate, given the imbalance between subgroups (11AMS vs. 9 non‐AMS) and the non‐parametric nature of some of the biomarkers.

Preliminary classification models developed from overnight recordings at 2600 and 3647 m predicted AMS at 4554 m with ‘good’ accuracy (100% and 80%, respectively) and exhibited ‘favourable’ sensitivity (i.e., a high proportion of true positives and low proportion of false negatives; 100% and 90.9%, respectively; Appendix 2). However, the 100% accuracy signal presents methodological issues, and indicates that additional measures were required to prevent overfitting and/or data leakage during cross‐validation steps. Nevertheless, the methodologies outlined here might still serve as a starting point for future modelling.

### 4.3 Limitations and future directions

The limited sample size of the present study, a common problem encountered with such field‐based research studies (Ainslie, 2014), is a clear limitation and limits the ability to draw definitive conclusions related to biomarkers of importance. In addition, the small sample size could have resulted in overfitting with the model; however, the present study was designed as a pilot study. These results provide a foundation for future studies and might provide an insight into the biomarkers that might prove useful for future classification model optimisation. Likewise, they highlight the need for larger overnight oximetry field studies.

A strength of this study is that clinical examination, conducted by a General Medical Council registered consultant physician, was used as the end‐point criterion for AMS categorization, which helped to limit any confound owing to the subjectivity of the LLS.

This model remains applicable only to the ascent profile embodied in this study. As suggested in a recent review paper, there is a need to implement a factor within prediction models related to the altitude rate of ascent within the preceding days/hours, because this is likely to impact the propensity/risk for illness greatly (Goves et al., 2024). Future studies working to develop a model that is applicable across altitudes and rates of ascent, or studies aiming to form a database from previous expedition data in this pursuit, should standardise the method used to define altitude and rate of ascent before factoring this into the model. Likewise, the present model(s) are applicable only to individuals who do not have a history of any prophylactic drug use (e.g., acetazolamide). Future studies must consider this factor (i.e., prophylactic acetazolamide use) and how this might impact relativity/applicability of the findings, particularly given that this is likely to be a common occurrence among mainstream/commercial trekking groups.

This pilot study was not intended to be exhaustive in the degree of included biomarkers but to provide a foundation for future research in this area and to contribute to knowledge synthesis for altitude studies. As such, we recognise that there are likely to be additional overnight oximetry biomarkers that could improve the predictive model performance. For example, the delta index (Δ index) of overnight $S_{pO_{2}}$, which has been shown to improve detection of apnoea‐related sleep disturbances (Pépin et al., 1991), could be useful to include, notwithstanding the complexities surrounding sleep disturbances and the association with altitude sickness (Scherrer & Verges, 2017). Nevertheless, it displays areas of improvement with a range of additional biomarkers for consideration from the $S_{pO_{2}}$ signal. Likewise, future studies would benefit from evaluation of additional biomarkers from the HR signal, including HR variability (time, frequency and non‐linear domains; Boos et al., 2018). Given that the present study was built largely around existing biomarkers, specifically in relationship to PSD, it remains possible that alternative frequency bands might be more relevant and sensitive for the detection and prediction of AMS; subsequent studies might aim to explore this. Future similar analysis might also consider alternative or additional methods for the comparison of classification models (e.g., Area Under the Receiver Operating Characteristic Curves). These studies should also consider analysis of the overnight biomarkers in a way that enables the prediction of AMS in relationship to symptom severity (e.g., from LLS).

## 5 CONCLUSION

Time‐derived, frequency‐derived and non‐linear biomarkers extracted from overnight oximetry recordings were useful in demonstrating the $S_{pO_{2}}$ morphologies during ascent and demonstrated clear physiological linkage. Preliminary classification models trained using overnight oximetry biomarkers were predictive of AMS occurring at 4554 m (as determined by clinical examinations) in this pilot study; however, the limited sample size meant that modelling results must be approached with caution. Additional research is required to evaluate these and other oximetry biomarkers in a larger cohort and to produce more robust predictive models.

## AUTHOR CONTRIBUTIONS

Each author has read and approved the final version of this manuscript and agrees to be accountable for all aspects of the work. Each person designated as an author qualifies for authorship.

## CONFLICT OF INTEREST

None declared.

## GENERATIVE AI STATEMENT

The authors confirm that no generative artificial intelligence tools, large language models, or AI‐assisted technologies were used in the drafting, data analysis, or generation of materials for this manuscript.

## Data Availability

Data are available from the corresponding author upon reasonable request.

## APPENDIX 1

### METHODS FOR EXPLORATORY ANALYSIS RELATED TO FEATURE SELECTION, CLASSIFICATION TRAINING/VALIDATION AND MODEL PERFORMANCE

#### Feature selection

To avoid overfitting in the context of a small sample size, visual inspection of the correlation heatmap was used initially to remove one feature from each pair of highly correlated biomarkers (with Pearson's *r* = > 0.95). A hierarchical approach was then adopted for remaining biomarkers based on our understanding of the existing literature in addition to empirical evidence from previous field expeditions. Following this, a systematic approach was applied to remaining biomarkers across multiple supervised filter‐based feature selection methods [e.g., minimum redundancy maximum relevance (MRMR) and χ^2^, Kruskal–Wallis *H* test], with the three highest ranking (and most dissimilar) biomarkers chosen for each feature selection method. To avoid redundancy, only the highest‐ranking TST $S_{pO_{2}}$ metric was chosen when multiple TST $S_{pO_{2}}$ metrics were ranked amongst the top three biomarkers. Classification models were then trained and cross‐validated for each of the groups of three biomarkers from each selection method using MATLAB (v.2022a, MathWorks, Natick, MA, USA), as described below.

#### Classification training/validation (binary classification)

Models were trained with a 5‐fold (*k*‐fold) cross‐validation method to protect against overfitting. Classification models were trained across the biomarkers chosen from the various feature selection methods to determine the optimal combination of both biomarkers and model type, similar to methods previously described (Alvarez et al., 2013).

A variety of available classification models were applied using MATLAB and included: support vector machines (SVMs; linear, quadratic, cubic, fine Gaussian, medium Gaussian and coarse Gaussian), nearest neighbour classifiers (fine, medium, coarse, cosine, cubic and weighted), kernel approximation classifiers (SVM and logistic regression), ensemble classifiers (boosted trees, bagged trees, subspace discriminant, subspace KNN and RUSBoosted trees), decision trees (fine, coarse and medium), discriminant analysis (linear and quadratic), logistic regression classifiers, naive Bayes classifiers (Gaussian and kernel) and neural network classifiers (narrow, medium, wide, bilayered and trilayered).

Model performances (accuracies) were then compared between models as described in the next subsection, with the highest‐performing model chosen for comparison to the highest‐performing models of other nights of ascent.

#### Model performances and comparison

Classification models were evaluated based on predictions for the occurrence of AMS at 4554 m (on either the first or second day). Model performances (sensitivity/TPR, specificity and accuracy) were evaluated and compared for each night, with the highest‐performing model (ranked based on accuracy of the testing/validation) for each set of three biomarkers across the different feature selection methods used in the comparison. The highest‐performing model for each night was then compared between other nights of ascent based on model performances to determine which, if any, individual altitude could be used to predict AMS prospectively. For models with equal accuracy, the model with the higher sensitivity (or TPR; higher true‐positive and lower false‐negative rates) was preferred, given the clinical implications of a false negative in the context of AMS in the field.

## APPENDIX 2

### CLASSIFICATION MODEL PERFORMANCES FROM EACH NIGHT OF ASCENT (MARGHERITA HUT) BASED ON THREE DIFFERENT FEATURE SELECTION METHODS, EACH INCLUDING TWO TO THREE BIOMARKERS

**Table:**

Feature selection method + model	Biomarkers	Results from Confusion Matrix				Sens/TPR (%)	Spec/TNR (%)	Accuracy (%)
		TP	TN	FP	FN			
**Gressoney, 1823 m (*n* = 20)**								
MRMR (3 biomarkers) + KNN	TST78 Lowest desat $S_{pO_{2}\max}$	7	4	5	4	63.6	44	55%
Kruskal–Wallis *H* (3 biomarkers) + fine, medium, coarse trees	MF *Mf3* (kurtosis) TST78	8	5	4	3	72.7	44	65
χ^2^ (3 biomarkers) + KNN	*Mf2*_band_ (variance) PF TST78	10	6	1	3	90.9	85.7	80
**Orestes Hütte, 2600 m (*n* = 16)**								
MRMR (2 biomarkers)^*^ + SVM	$S_{pO_{2}\max}$ TST77	7	7	0	2	77.8	100	87.5
Kruskal–Wallis *H* (3 biomarkers) + KNN	LCZ HR_min_ MF	9	7	0	0	100	100	100
χ^2^ (3 biomarkers) + fine, medium, coarse trees	$S_{pO_{2}\max}$ LCZ ApEnt	9	5	0	2	81.8	100	87.5
**Gnifetti Hut, 3647 m (*n* = 20)**								
MRMR (3 biomarkers) + linear discriminant or SVM	PF $S_{pO_{2}\max}$ TST64	8	7	2	3	72.	77.8	75
Kruskal–Wallis H (3 biomarkers) + SVM or Subspace	PF Δ1^st^–2^nd^ half HR/$S_{pO_{2}}$	9	7	2	2	81.8	77.8	80
χ^2^ (3 biomarkers) + SVM	HR events/h							
3 biomarkers	TST84	10	6	3	1	90.9	66.7	80
2 biomarkers	ApEnt	10	6	3	1	90.9	66.7	80

*Note*: The number of analysable recordings (*n*) obtained at each altitude is noted under the altitude/location. Four recordings were unrecoverable at Orestes Hütte, resulting in *n* = 16. Models were trained for predicting AMS (by clinical examination) occurring anytime during the stay at 4554 m.

Abbreviations: ApEnt, approximate entropy; FN, false negative; FP, false positive; KNN, *k*‐nearest neighbour; LZC, Lempel–Ziv complexity; MF, median frequency; MRMR, minimum redundancy, maximum relevance; PF, peak frequency; Sens, sensitivity; SVM, support vector machine; TP, true positive; TPR, true positive rate; TN, true negative; TSTx or CTx, total saturation time or cumulative time at ‘x’ peripheral O_2_ saturation.

^*^Only two non‐correlated dissimilar biomarkers could be chosen from highest ranking biomarkers (i.e., multiple TST in top ranked values).

## APPENDIX 3

### VISUAL REPRESENTATION OF THE SEPARATION BETWEEN ACUTE MOUNTAIN SICKNESS SUBGROUPS (AMS VS. NON‐AMS)

(a) Predictions of AMS (occurring at 4554 m) were made using the KNN classification (with Kruskal–Wallis *H* feature selection) for overnight oximetry data from Orestes Hütte (2600 m). Three biomarkers were selected for this model although, for simplicity, only two biomarkers (LZC vs. median frequency) are plotted here. (b) These groupings are compared with the same biomarkers from Gnifetti Hut (3647 m). Abbreviation: AMS, acute mountain sickness; LZC, Lempel–Ziv complexity.
